# “Bigger” or “better”: the roles of magnitude and valence in “affective bias”

**DOI:** 10.1080/02699931.2019.1662373

**Published:** 2019-09-09

**Authors:** Jack Love, Oliver J. Robinson

**Affiliations:** Neuroscience and Mental Health group, Institute of Cognitive Neuroscience and Research Department of Clinical, Educational and Health Psychology, University College London, London, UK

**Keywords:** Affective bias, two-alternative forced choice, anxiety, valence, magnitude

## Abstract

Negative affective biases are thought to be a key symptom driving and upholding many psychiatric disorders. When presented with ambiguous information, anxious individuals, for example, tend to anticipate lower rewards than asymptomatic individuals (Aylward et al., 2019. Translating a rodent measure of negative bias into humans: the impact of induced anxiety and unmedicated mood and anxiety disorders. *Psychological Medicine*). The assumption is that this is because anxious individuals assume “worse” outcomes. However, predictions are often made about high and low rewards, so it is not clear whether the bias is due to the valence (the “worse” option) or just magnitude (the lower number). We therefore explored the roles of valence and magnitude in a translational measure of negative affective bias. We adapted a two-alternative forced choice (2AFC) “reward-reward” task into a “punishment-punishment” paradigm, and followed up with “high reward-high punishment” and “low reward-high punishment” variants. The results from the “punishment-punishment” paradigm – a bias *towards higher punishments* in healthy controls – suggest that it is outcome *magnitude* that is important. However, this is qualified by the other variants which indicate that both valence and magnitude are important. Overall, our results temper the assumption that negative affective biases observed in tasks using numeric outcomes are solely as a result of subjective outcome valence.

Negative affective bias refers to the phenomenon whereby patients with mood disorders, such as anxiety and depression, but also many other psychiatric disorders, tend to prioritise emotionally negative or unfavourable information or outcomes (Mathews & MacLeod, 1994). This bias can reveal itself in many ways. Anxious and depressed patients appear “pessimistic” in tasks which require them to anticipate the probability of a reward (Pizzagalli, Iosifescu, Hallett, Ratner, & Fava, 2008); identify a higher proportion of unfavourable words than neutral words (Foa & McNally, 1986; Powell & Hemsley, 1984); have longer response latencies to respond to words with emotionally-negative connotations (Gotlib & McCann, 1984; Watts, McKenna, Sharrock, & Trezise, 1986); and are able to more rapidly identify attentional probes when they appear in the region of a negative word (Broadbent & Broadbent, 1988; MacLeod, Mathews, & Tata, 1986).

Although many cognitive tasks used to investigate affective states utilise verbal or visual stimuli (e.g. faces), a large number use numeric outcomes. This may take the form of a “reward-reward” (R-R) two-alternative forced choice (2AFC) task, where the participant is required to estimate whether an ambiguous tone is closer in frequency to a tone associated with a higher reward or one associated with a lower reward (Aylward, Hales, Robinson, & Robinson, 2019). The prediction is that those afflicted by a negative affective bias will tend to anticipate lower rewards more often than higher rewards. The problem with this paradigm, however, is that is rests on the assumption that lower rewards are chosen because they are intrinsically lower in value than the higher rewards, rather than simply a lower magnitude. For example, Brilot, Asher, and Bateson (2010) explored the association between stereotypic behaviour and “negative affect” in starlings, using food rewards of differing sizes. The fact that birds which somersaulted more were more likely to anticipate a low reward was taken to mean that somersaulting was symptomatic of a negative affective state. Hales, Robinson, and Houghton (2016) also found a preference for the low reward when rats were subject to either acute drug-induced anxiety or chronic restraint stress, again with the assumption that this indicated a negative affective state. Most recently, Aylward et al. (2019) used a similar 2AFC task and found that humans suffering from anxiety mimic the choice behaviour of rodents undergoing anxiogenic manipulation. The translational nature of the task, between animals and humans, is useful, however it has not been demonstrated that this choice bias is due to valence (i.e. the subjective, positive or negative, value of the outcome) rather than magnitude (i.e. the absolute size of the outcome, whether a reward or a punishment). Although valence and magnitude are equivalent in tasks which offer two different monetary rewards, in the real world, a “bigger” car is not always the “better” car, for instance.

To differentiate between these hypotheses, we expanded the investigation to include a “punishment-punishment” (P-P) or “negative domain” 2AFC task, where participants must choose whether they anticipate a larger or a smaller monetary loss. Presumably, a bias towards both larger rewards and punishments (i.e. a “magnitude bias”) could signify a bias towards more “extreme” outcomes in general. This is not implausible: the environment in which we live is noisy, and the brain must be able to filter out relatively unimportant information whilst being alerted to “important” inputs, regardless of whether they are perceived to be “positive” or “negative”. One theory addressing such a phenomenon is known as the “salience bias” or “perceptual salience”, and is well-documented in the literature (Bordalo, Gennaioli, & Shleifer, 2012; Tversky, Slovic, & Kahneman, 1982).

However, we predicted that outcome valence would be the deciding factor, and as a bias towards “positive” outcomes has been demonstrated in healthy individuals using different tasks (Anderson, Hardcastle, Munafò, & Robinson, 2011; Erickson et al., 2005), we expected to see a bias towards the lowest punishment in an unselected sample. Contrary to predictions, the effect appeared to be driven by outcome magnitude (and those who anticipated higher gains also anticipated higher losses), so we then explored bias on follow-up “high reward-high punishment” and “low reward-high punishment” task variations. We predicted that if the effect was entirely due to magnitude, we should see no bias towards either outcome on the first task (where magnitudes are matched), and a bias towards losses on the second task (because the gain has a lower magnitude). We then performed a full replication based on effect size estimates from the initial studies. Finally, we isolated the negative domain task from experiment 1 and replicated a third time in the absence of reward stimuli, to demonstrate that effects are not specific to the original (task-switching) context.

## Method

### Study design

We used the online behavioural science platform Gorilla (https://gorilla.sc/) to conduct the tasks. We used, and modified, an existing R-R 2AFC task in our assessment of cognitive bias. 2AFC tasks have originally been conducted using an auditory platform for stimulus presentation, although it has been demonstrated that they can be translated onto a visual platform whilst retaining the same sensitivity to the affective bias. For example, a repeated measures ANOVA found no effect of task on the anticipation of favourable outcome (*F*_(2102)_ = 1.357, *p* = 0.262) (Jaber Ansari, 2017), which is the key measure of affective bias on this task. Using a visual platform (where participants must decide whether a shape is closer in size to one associated with a higher or lower reward) is more appropriate for online platforms as it removes any confounding factors associated with speaker or headphone availability.

### Participants

Experiment 1 involved 45 participants. We powered experiment 2 (*N* = 67) to detect an effect size *d* = 0.347 to match the effect size found in experiment 1, for the same measure as experiment 1, at *p* < 0.05 with 80% power. After these experiments, we replicated our analyses in a larger sample size for both experiment 1 (*N* = 122) and experiment 2 (*N* = 130). This allowed us to detect a small-medium effect size *d* = 0.3 at *p* < 0.05 with 90% power. Statistical inference was run on the two experiments separately. However, to get more accurate estimates of effect sizes we pooled these results across experiment 1 (*N* = 167) and experiment 2 (*N* = 197). Finally, we isolated the negative domain task from experiment 1 and replicated this task again, this time using 156 participants on the basis of the average pooled effect sizes we observed across experiments 1 and 2.

Participants were recruited online in the Amazon Mechanical Turk (MTurk) marketplace, and completed a consent form prior to taking part (UCL ethics reference 6199/001). Aside from access to a computer or laptop with an internet connection, there were no specific exclusion criteria for the study. Participants were able to withdraw from the study at any time by closing their browser, and randomisation of task order proceeded according to participant recruitment number and Gorilla's built-in randomisation procedure. Our data are available at https://osf.io/uxcd7/.

### Details of the tasks

**Figure 1.**
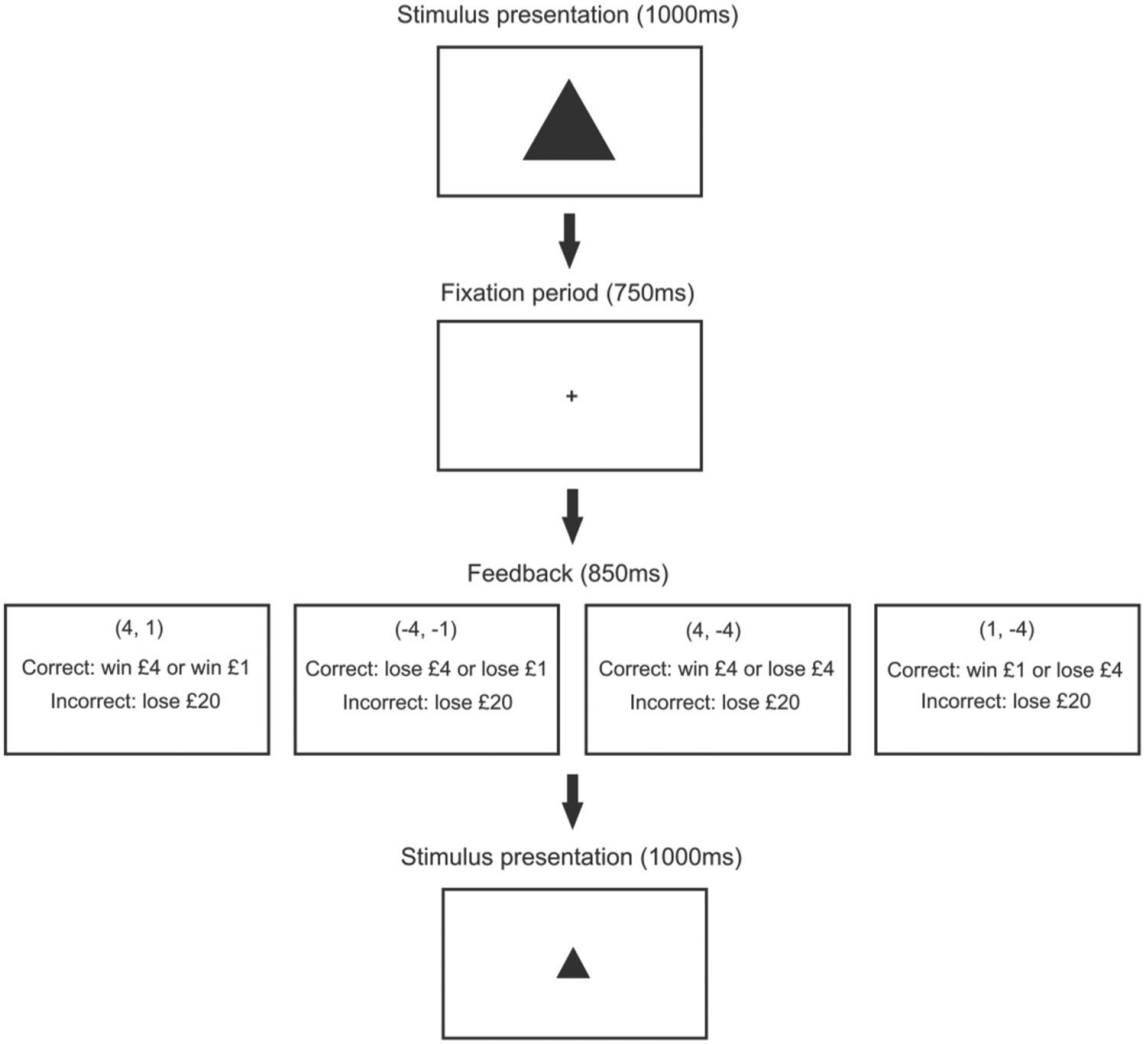
A schematic of the method used. The feedback outcomes are shown for each task.

A schematic of both experiment 1 and experiment 2 is shown in Figure 1.

### Experiment 1

Experiment 1 consisted of an R-R task and a P-P task. Participants completed both tasks in a randomly-allocated order. Both main tasks were preceded by a practice block. In the practice block, participants were presented with a random series of big (400 × 400) and small (200 × 200) shapes, and had to press the “z” and “m” keys on the keyboard to identify whether a shape was big or small.

In the R-R task, responding correctly to the shape resulted in a big (£4) or small (£1) reward, depending on the size of the shape. An incorrect or absent response resulted in a £20 loss. Participants started the task with no money and were told to try and win as much money as possible. The Hales et al. (2016) and Aylward et al. (2019) studies both used values of 1 and 4 as rewards, and as biases in choice behaviour were able to be detected using such values, we have used the same ones in the current study.

In the P-P task, responding correctly to the shape resulted in a big (-£4) or small (-£1) loss, depending on the size of the shape. An incorrect or absent response led to a £20 loss, in order to discourage abstinence from responding. Participants were told they would start with £800 and were instructed to try and minimise their losses.

Stimuli were presented for 1000 ms, followed by a 750 ms fixation period. Therefore, the participants had 1750 ms in total to respond. Feedback on the response was then presented for 850 ms. In the practice block, 10 big shapes and 10 small shapes were presented.

The main task followed the same structure as the practice block. However, as well as the “unambiguous” 400 × 400 and 200 × 200 shapes, participants were shown “ambiguous” mid-size (300 × 300) shapes, which randomly corresponded to either outcome. In the main task, there were 120 trials, with 40 big shapes, 40 small shapes and 40 mid-size shapes being randomly presented.

### Experiment 2

We ran a second experiment to build on the results we attained in experiment 1, and further tease apart the effects of valence and magnitude. Keeping the basic structure of both tasks the same, we changed the outcomes in the positive domain task to a £4 win and a £4 loss (4, −4), and those in the negative domain to a £1 win and a £4 loss (1, −4). This resulted in a “high reward-high punishment” task, and a “low reward-high punishment” task. As before, participants lost £20 if they gave an incorrect answer.

### Statistical analyses

Statistical analyses were run using JASP (0.8.5.1). Choice behaviour was assessed by calculating the proportion of ambiguous stimuli that were interpreted as a “favourable outcome” (*p*(mid = favourable)). This was analysed using a two-tailed one-sample *t*-test with a test value of 0.5, and a within-subject *t*-test. A *valence* bias would cause *p*(mid = favourable) to be significantly above 0.5 in all experiments.

Correlation analyses were run on *p*(mid = favourable) between tasks. A valence bias would result in a positive correlation between the anticipation of the favourable outcome in each task. Independent *t*-tests were also run to compare choice behaviour across the two experiments (i.e. between the (4, 1) and (4, −4), and (4, 1) and (1, −4) tasks), in order to see whether increasing the magnitude of the unfavourable outcome also increased the bias towards it. If bias was driven by magnitude, we would expect this to be the case.

We also collected accuracy and reaction time measures for each outcome on each task.

In our replications we used one-tailed instead of two-tailed *t*-tests whenever this was justified based on clear predictions from previous results. In the dataset pooled across initial and replication studies, we do not calculate inferential statistics, only effect sizes.

Bayesian statistics were also used; Bayesian inference compares the likelihood of observing the given data under the alternative hypothesis (*H*_1_) with the likelihood of observing it under the null hypothesis (*H*_0_). *BF*_10_ refers to the evidence for *H*_1_ relative to *H*_0_, and can be graded, for example, as anecdotal (1–3), moderate (3–10), or strong (10–30). *BF*_+0_ and *BF*_−0_ refer to one-tailed *t*-tests where the hypothesis is that the bias is more than or less than 0.5 respectively. The Bayesian credible interval (BCI) is reported for *p*(mid = favourable), and is the range of values within which the true value lies with 95% probability. In all cases we used the JASP default Cauchy prior for these Bayesian analyses.

In both experiments, circular and triangular stimuli were counterbalanced across tasks to minimise learning being carried over between tasks. We counterbalanced the shape size and reward/loss contingencies, and the “z” and “m” key response and stimulus size contingencies, across participants (these were consistent across the two tasks for each participant). An ANOVA found a significant effect of the counterbalancing condition upon choice behaviour in the (4, 1) (*F*_(151)_ = 3.127, *p* < 0.001), (−4, −1) (*F*_(146)_ = 3.495, *p* < 0.001), (4, −4) (*F*_(181)_ = 4.944, *p* < 0.001), and (1, −4) (*F*_(175)_ = 3.474, *p* < 0.001) tasks. However, since the counterbalancing was matched for each participant across tasks, counterbalancing cannot account for any differences in task performance within each experiment.

## Results

### Experiment 1

#### Choice behaviour

##### Initial sample

**Figure 2.**
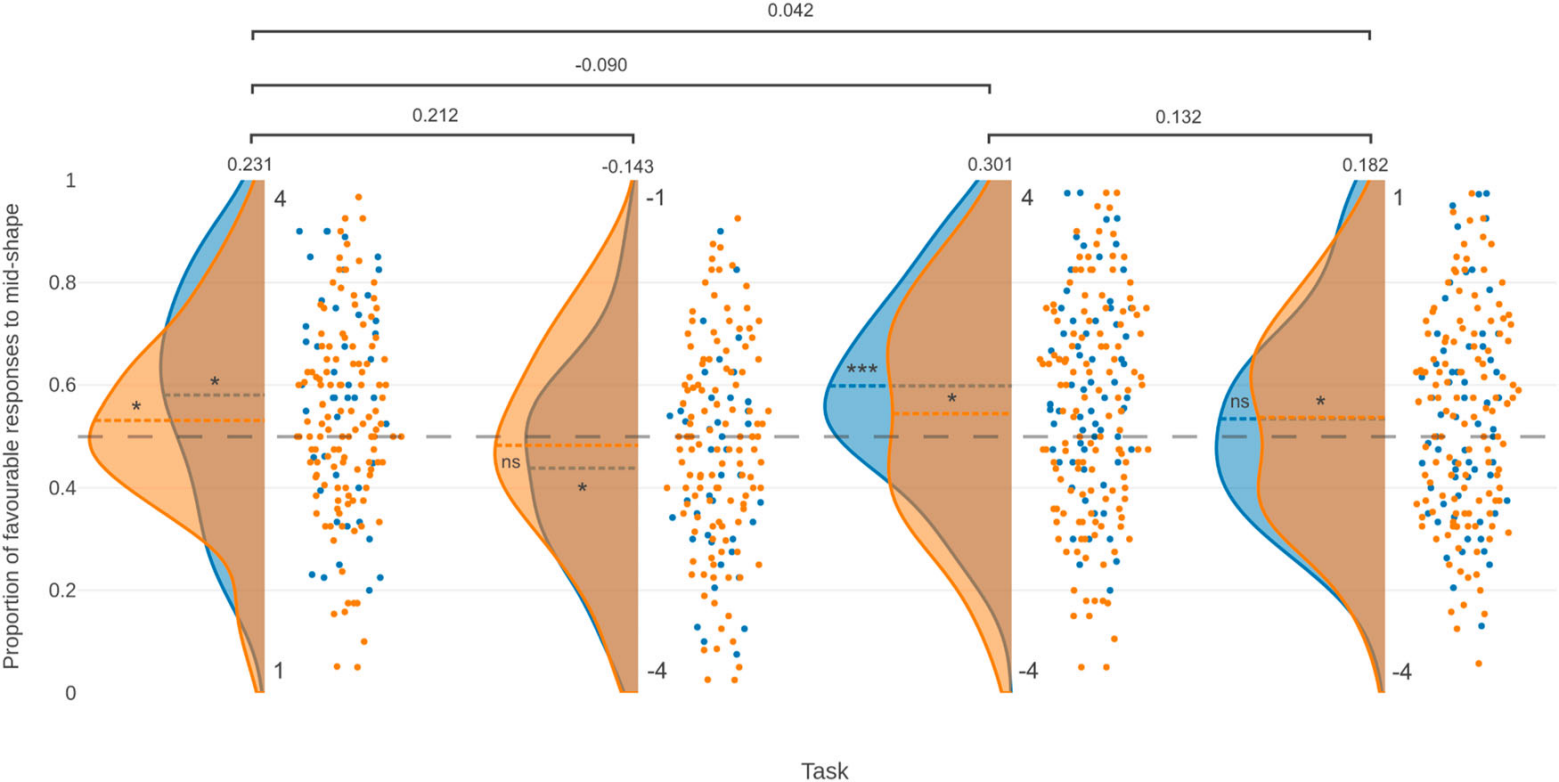
Raincloud plot showing the proportion of favourable responses to the mid-shape on the (4, 1) (*N* = 167), (−4, −1) (*N* = 162), (4, −4) (*N* = 197), and (1, −4) (*N* = 191) tasks, for the initial (blue) and replication (orange) samples. The dashed grey line represents a bias measure of 0.5, and the dashed coloured lines in each data set correspond to the mean line for that set. Significance levels for the bias measures are shown for each sample (* *p* < 0.05, ***p < 0.001 for test against 0.5; ns = not significant). Effect sizes are shown for the pooled sample for each task, and for the pooled differences in the bias measure between tasks.

In the positive domain task, participants were more likely to anticipate a high reward (i.e. 4 over 1; *t*_(44)_ = 2.580, *p* = 0.013, *d* = 0.385, *BF*_10_ = 3.061, BCI [0.518, 0.643]), and in the negative domain task they were more likely to anticipate a high punishment (i.e. −4 over −1; *t*_(41)_ = −2.103, *p* = 0.042, *d* = −0.325, *BF*_10_ = 1.217, BCI [0.379, 0.498]). There was a significant difference in bias towards the favourable outcome between the two tasks (*t*_(41)_ = 2.674, *p* = 0.005, *d* = 0.413, *BF*_+0_ = 7.499), but when the bias score was reversed in the P-P task so that the extent of the bias towards 4 and −4 in the two tasks could be compared, there was no significant difference between choice probability across the two tasks (*t*_(41)_ = 0.881, *d* = 0.136, *p* = 0.192); and Bayesian analysis favoured the null model (*BF*_+0_ = 0.385), i.e. that the bias measure is equivalent between 4 and −4. In other words, participants favoured the larger (rather than more favourable) of the two options in both cases. This is presented in Figure 2.

##### Replication sample

As before, participants were more likely to anticipate a high reward (*t*_(121)_ = 1.866, *p* = 0.032, *d* = 0.169, *BF*_+0_ = 1.042, BCI [0.498, 0.565]). However, in the negative domain task there was no significant bias in either direction (*t*_(119)_ = −0.923, *p* = 0.179, *d* = −0.084, BF_−0_ = 0.251, BCI [0.446, 0.520]). There was no significant difference in bias towards the favourable outcome between the two tasks (*t*_(114)_ = 1.461, *p* = 0.073, *d* = 0.136, *BF*_+0_ = 0.537). Again, however, there was no significant difference between the bias towards 4 and that towards −4 (*t*_(114)_ = 0.854, *p* = 0.198, *d* = 0.080, *BF*_+0_ = 0.236). This is presented in Figure 2.

##### Pooled samples

*p*(mid = favourable) was above 0.5 in both the positive domain (0.545 (SD 0.194), *t*_(166)_ = 2.983, *d* = 0.231) and below 0.5 in the negative domain (0.471 (SD 0.199), *t*_(161)_ = −1.824, *d* = −0.143).

All data collected for the initial, replication and pooled samples in experiment 1, including accuracy and reaction time measures, are displayed in Table 1.

**Table 1.** Summary of the key measures in experiment 1. For each measure, the results from the initial, replication and pooled samples are shown.

Task	Outcome	Initial				Replication				Pooled			
		Bias (%) (SD)	Accuracy (%) (SD)	Reaction time (ms) (SD)	Reaction time to ambiguous shape (ms) (SD)	Bias (%) (SD)	Accuracy (%) (SD)	Reaction time (ms) (SD)	Reaction time to ambiguous shape (ms) (SD)	Bias (%) (SD)	Accuracy (%) (SD)	Reaction time (ms) (SD)	Reaction time to ambiguous shape (ms) (SD)
(4, 1)	Favourable	0.580 (0.209)	0.924 (0.108)	594 (181)	685 (199)	0.532 (0.187)	0.830 (0.219)	581 (247)	638 (256)	0.545 (0.194)	0.855 (0.199)	585 (230)	651 (242)
(4, 1)	Unfavourable	0.420 (0.209)	0.912 (0.157)	608 (180)	699 (198)	0.468 (0.187)	0.835 (0.207)	583 (239)	651 (265)	0.455 (0.194)	0.855 (0.197)	589 (224)	664 (249)
(−4, −1)	Favourable	0.438 (0.190)	0.951 (0.0709)	658 (223)	723 (250)	0.483 (0.202)	0.835 (0.204)	595 (234)	668 (242)	0.471 (0.199)	0.865 (0.186)	612 (232)	682 (245)
(−4, −1)	Unfavourable	0.562 (0.190)	0.942 (0.0805)	644 (189)	751 (217)	0.517 (0.202)	0.822 (0.220)	596 (233)	654 (263)	0.529 (0.199)	0.853 (0.201)	609 (223)	679 (255)

##### Isolated negative domain task

**Figure 3.**
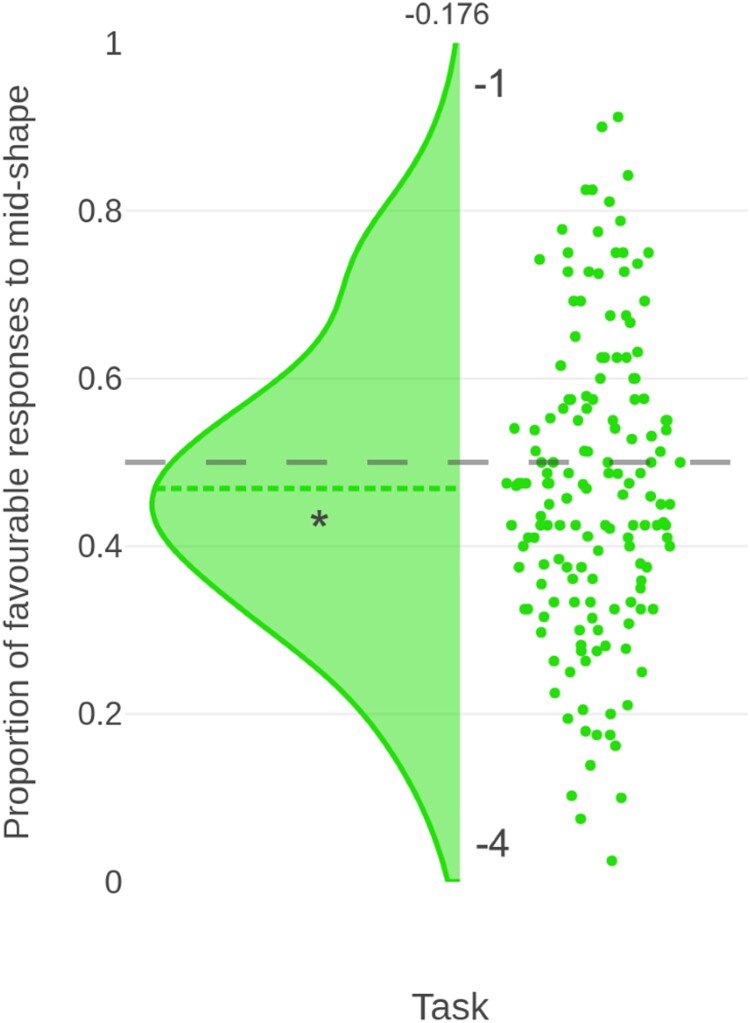
Raincloud plot, using the same layout as Figure 2, showing the proportion of favourable responses to the mid-shape on a second replication of the (−4, −1) (*N* = 156) task on its own (i.e. isolated from any reward stimuli).

Here, we presented the (−1, −4) (negative domain) task on its own, in order to remove the effects of any reward stimuli. As in the initial sample, on full-powered second replication participants were more likely to anticipate a high punishment than a low punishment in the negative task (*t*_(155)_ = −2.196, *p* = 0.030, *d* = −0.176, *BF*_10_ = 0.920, BCI [0.441, 0.497]). The bias measure from the isolated negative domain task is demonstrated in Figure 3*.*

#### Correlations

**Figure 4.**
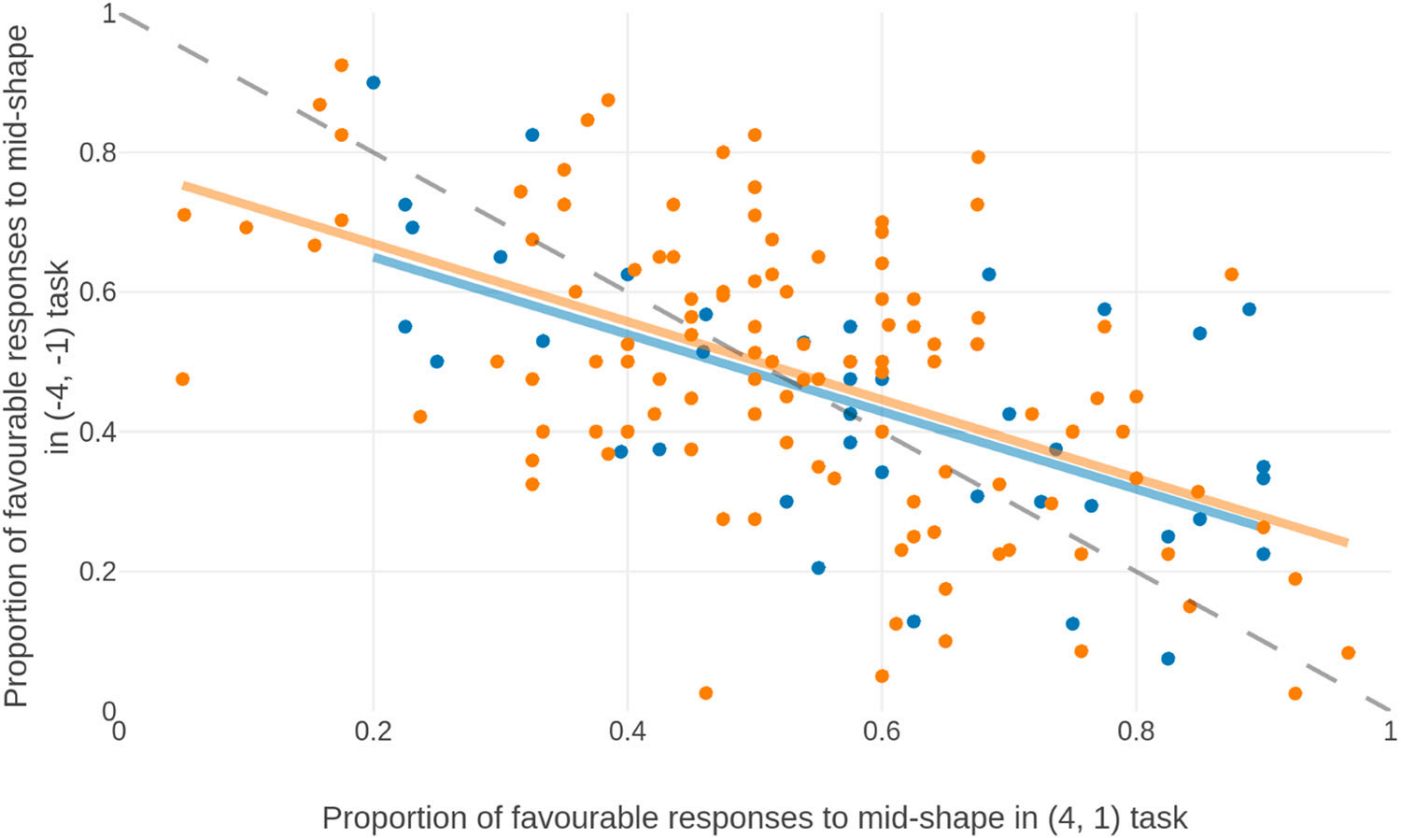
Correlation between the proportion of high outcome responses to mid-shapes in the positive and negative domains, in both the initial sample (blue) and replicated sample (orange). The negative correlation indicates that individuals who chose the most favourable option in the positive domain chose the least favourable option in the negative domain, indicating that they chose on the basis of magnitude rather than valence. The dashed grey line is the identity.

There was a negative correlation between *p*(mid = favourable) across both domains in the *initial* (*r* = −0.598, *p* < 0.001, *BF*_10_ > 30), *replication* (*r* = −0.523, *p* < 0.001, *BF*_10_ > 30) and *pooled* (*r* = −0.548) samples, suggesting that those who showed a bias towards high rewards also showed a bias towards high punishments and vice versa. This correlation is shown in Figure 4.

### Experiment 2

#### Choice behaviour

##### Initial sample

In the (4, −4) task, there was a significant bias towards the reward (*t*_(66)_ = 4.301, *p* < 0.001, *d* = 0.525, *BF*_10_ > 30, BCI [0.553, 0.644]). In the (1, −4) task, there was no significant bias towards either outcome (*t*_(64)_ = 1.415, *p* = 0.162, *d* = 0.175, *BF*_10_ = 0.351, BCI [0.486, 0.584]). In this case, the bias towards the reward was significantly higher in the (4, −4) task than it was in the (1, −4) task (*t*_(63)_ = 2.307, *p* = 0.012, *d* = 0.288, *BF*_10_ = 1.599). This is presented in Figure 2.

##### Replication sample

There was a significant bias towards the reward in both the (4, −4) task (*t*_(129)_ = 2.345, *p* = 0.010, *d* = 0.206, *BF*_+0_ = 2.694, BCI [0.507, 0.583]) and the (1, −4) task (*t*_(125)_ = 2.076, *p* = 0.040, *d* = 0.185, *BF*_10_ = 0.788, BCI [0.502, 0.573]). There was no significant difference between the bias in the (4, −4) task and in the (1, −4) task (*t*_(122)_ = 0.470, *p* = 0.320, *d* = 0.042, *BF*_+0_ = 0.151). This is presented in Figure 2.

##### Pooled samples

There was a numeric bias towards the reward in both the (4, −4) task (0.563 (SD 0.209), *t*_(196)_ = 4.228, *d* = 0.301) and the (1, −4) task (0.536 (SD 0.200), *t*_(190)_ = 2.519, *d* = 0.182), albeit with a considerably reduced effect size.

All data collected for the initial, replication and pooled samples in experiment 2, including accuracy and reaction time measures, are displayed in Table 2.

**Table 2.** Summary of the key measures in experiment 2. For each measure, the results from the initial, replication and pooled samples are shown.

Task	Outcome	Initial				Replication				Pooled			
		Bias (%) (SD)	Accuracy (%) (SD)	Reaction time (ms) (SD)	Reaction time to ambiguous shape (ms) (SD)	Bias (%) (SD)	Accuracy (%) (SD)	Reaction time (ms) (SD)	Reaction time to ambiguous shape (ms) (SD)	Bias (%) (SD)	Accuracy (%) (SD)	Reaction time (ms) (SD)	Reaction time to ambiguous shape (ms) (SD)
(4, −4)	Favourable	0.598 (0.187)	0.875 (0.181)	577 (206)	644 (218)	0.545 (0.218)	0.819 (0.215)	525 (245)	597 (278)	0.563 (0.209)	0.838 (0.206)	543 (233)	613 (260)
(4, −4)	Unfavourable	0.402 (0.187)	0.814 (0.245)	593 (213)	647 (216)	0.455 (0.218)	0.751 (0.269)	547 (257)	577 (287)	0.427 (0.209)	0.772 (0.262)	563 (243)	601 (266)
(1, −4)	Favourable	0.535 (0.198)	0.857 (0.197)	609 (219)	664 (233)	0.537 (0.202)	0.793 (0.227)	525 (246)	567 (275)	0.537 (0.200)	0.815 (0.219)	554 (240)	600 (265)
(1, −4)	Unfavourable	0.465 (0.198)	0.828 (0.232)	620 (221)	677 (250)	0.463 (0.202)	0.735 (0.261)	541 (264)	557 (297)	0.463 (0.200)	0.767 (0.255)	568 (252)	598 (287)

#### Correlations

There was a significant positive correlation between *p*(mid = favourable) across both tasks in *initial* (*r* = 0.384, *p* = 0.002, *BF*_10_ = 18.65) *replication* (*r* = 0.588, *p* < 0.001, *BF*_10_ > 30) and *pooled* (*r* = 0.521) samples, meaning that those who had a bias towards the reward in one task had a similar bias in the other.

#### Between-experiment analysis

##### Initial sample

Independent *t*-tests revealed that changing the outcomes from (4, 1) to (4, −4) had no significant effect on *p*(mid = favourable) (*t*_(110)_ = −0.474, *p* = 0.318, *d* = −0.091). Bayesian analysis favoured the null model (*BF*_−0_ = 0.303).

Changing the outcomes from (4, 1) to (1, −4) also had no significant effect on *p*(mid = favourable) (*t*_(108)_ = 1.160, *p* = 0.124, *d* = 0.225). Bayesian analysis favoured the null model (*BF*_+0_ = 0.645), so we could accept the null hypothesis. Therefore, although there was no significant outcome bias on the (1, −4) task, its results were not significantly different to the (4, 1) task.

##### Replication sample

Changing the outcomes from (4, 1) to (4, −4) had no significant effect on *p*(mid = favourable) (*t*_(250)_ = −0.517, *p* = 0.303, *d* = −0.065). Bayesian analysis favoured the null model (*BF*_−0_ = 0.217), so the null hypothesis was accepted.

Changing the outcomes from (4, 1) to (1, −4) also had no significant effect on *p*(mid = favourable) (*t*_(246)_ = −0.232, *p* = 0.408, *d* = −0.030). Bayesian analysis favoured the null model (*BF*_−0_ = 0.168).

The overall bias measures from the pooled studies, for both experiments 1 and 2, are shown in Figure 2.

## Discussion

In experiment 1, counter to predictions, those who were more likely to anticipate a high reward were *also* more likely to anticipate a high punishment. These results do not support the hypothesis that the interpretation bias is due to outcome valence; rather, the bias appears to be due to the absolute magnitude of the outcome, whether that outcome is positive or negative. In experiment 2, we therefore attempted to probe this further using a task comparing a gain and a loss of 4, which should demonstrate no bias if the effect was purely magnitude, and a task comparing a gain of 1 and a loss of 4, which should demonstrate a bias towards the loss if the effect was purely magnitude. However, in both cases a significant bias was seen towards the favourable (gain) condition, which indicates an effect of valence.

The R-R task in experiment 1 is consistent with the “positive” bias found among healthy individuals in previous tasks. However, the P-P version of the task contradicts this. Specifically, the individuals who were more likely to select the high reward option were also more likely to select the high punishment option. To try to explain this, the results could be approached from two different perspectives. The first perspective posits that valence, or the subjective value of an outcome, is the key modulator of bias. Results from the R-R (4, 1) task and both tasks in experiment two ((4, −4) and (1, −4)) fit this perspective, because there was a bias towards the most favourable outcome in all cases. Also, the results from the between-experiment analyses suggested that increasing the magnitude of the unfavourable outcome did not increase the bias towards it. The negative domain task may therefore contradict this because the task was simply misinterpreted; the unintuitive “damage limitation” paradigm of this task may have caused some confusion whereby participants thought that the aim of the task was to lose as much money as possible; notably, we did not see the same effect in the first replication of the negative domain task.

However, the second perspective is that outcome magnitude (the absolute size of the outcome, whether positive or negative) is at least partially responsible for the results. This perspective is supported by correlation analysis in experiment 1, where those who favoured higher rewards also favoured higher losses, as well as the initial P-P task where there was an overall bias towards the higher loss. Perhaps more importantly, when we isolated the negative domain task such that participants did not also complete the original positive domain task, we *again* saw a bias towards the higher loss. This reduces the likelihood that the results from the initial sample were a result of confusion between the P-P and R-R tasks. Similarly, the considerably reduced effect size of the “favourable” bias on the (1, −4) task in the pooled data (and lack of significant bias in the initial sample) relative to the (4, −4) task suggests that the higher magnitude of the loss relative to the gain may have “dragged down” the “favourable” bias. However, it should be noted that this difference is not significant in the replication sample. Moreover, if the effect was purely magnitude then we would have demonstrated no overall bias in the (4, −4) version and a bias towards losses in the (1, −4) version. Thus, there appears to be a combined effect of magnitude and valence which may change depending on the outcome values used. The effect that we see in the P-P task may therefore be because when the task is reframed in the loss domain (i.e. it is only possible to lose), participants shift to a decision-making strategy that makes them warier of larger losses and more likely to anticipate them, as opposed to taking advantage of any large rewards on offer, as seen in the R-R task. Of course, there still remains the possibility that the results are due to a misunderstanding of task instructions, but the results from the isolated task in a large sample demonstrate that this is not driven by the presence of a reward task, or confusion due to task-switching.

This study is important due to the extensive use of 2AFC tasks to assess cognitive affective bias in research. Tasks of this nature are popular because they are translational between species; the impact of a treatment upon task performance in other species can serve as a good predictor of its effects in humans. When these tasks follow a “reward-reward” paradigm, there is often no distinction between a bias towards a “more valuable” reward, and simply a “larger” reward. The results from our study do not definitively support the idea that subjective valence alone produces the biases observed in emotional 2AFC tasks, and may suggest a possible influence of outcome magnitude. This implies that caution may be warranted when interpreting the results of 2AFC tasks as an assessment of cognitive affective bias, with care taken to control for any possible effects of outcome magnitude upon the bias.

In terms of future directions, one area of interest might be to explore the impact of multipliers on the numbers; for example, is the effect exacerbated for 10 and 40 relative to 1 and 4? In order to reduce the risk of confusion on behalf of the participants whilst still isolating the effects of valence and magnitude, it may be sensible to compare biases across 2AFC tasks which use outcome combinations of (1, 2), (1, 3), (1, 4) and (1, 5), rather than contrasting two wins of different magnitudes against two losses of different magnitudes. It could also be interesting to move away from the 2AFC design and explore choice behaviour when using outcomes of 4, 1, −1 and −4 in the same task. A similar paradigm may also be used in other tasks adapted to probe affective biases which use non-numeric outcomes or stimuli. For example, the emotional Stroop task requires subjects to name the colour of an emotionally-valenced word, with the rationale being that longer response latencies signify an increased attentional bias to the word. Response times of healthy participants to “weakly positive” versus “strongly negative” words in a task such as this could help to delineate the effects of the “valence” and the “magnitude” of a stimulus.

One potential limitation of our study stems from the online method of data collection, where it is difficult to standardise the surroundings and behaviour of participants whilst they complete the cognitive tasks. Although it has been shown that online data collection on MTurk leads to no more issues with task engagement than data collection in the lab (Thomas & Clifford, 2017), collecting data in person is the only feasible way to guarantee that external factors are kept constant between subjects and, if the effects are due to confusion about task instructions, is a better way to asses this possibility. Also, although prior work (Aylward et al., 2019) has linked such tasks to affective biases in psychopathology, it is important to note that this sample was not screened and we have no way of directly linking this effect to clinical symptoms. Future work should explore the relationship between these effects and clinically relevant symptoms. It should be noted that we had no a priori predictions about the influence of demographic variables on our effects of interest and as such did not acquire this data. Future work may address this limitation by exploring the impact of such variables on magnitude and valence bias.

## Conclusions

Behavioural experiments of this nature are an important first step towards understanding exactly how mood disorders and their treatments may operate, as they provide a rudimentary correlate of the way the brain processes information to interpret the world around it. However, these experiments will only prove to be useful if the conclusions we draw from them are valid, and this necessitates exploring the various factors that may affect decision-making in such a setting, including outcome magnitude. Only with a robust understanding of the cognitive traits exhibited by those with affective disorders can meaningful progress be made regarding their treatment and interventions.
